# Profuse coarse pulmonary nodules in a patient with lymphangioleiomyomatosis: thirty-three years of follow-up

**DOI:** 10.3402/ecrj.v1.26272

**Published:** 2014-12-09

**Authors:** Daniel B. Rasmussen, Saher B. Shaker, Niels Seersholm, Sara Colella, Paul F. Clementsen

**Affiliations:** Department of Respiratory Medicine, Gentofte University Hospital, Hellerup, Denmark

**Keywords:** Lymphangioleiomyomatosis, cysts, nodules, neoplasm

## Abstract

Lymphangioleiomyomatosis (LAM) is a rare disease characterized by progressive cystic destruction of the lungs. We present an unusual radiological presentation of lymphangioleiomyomatosis in a patient followed for 33 years with profuse coarse lung nodules in addition to the classical cystic lesions. We believe that this report might support the case for considering LAM a low-malignant neoplasm.

Lymphangioleiomyomatosis (LAM) is a rare disease characterized by proliferation of abnormal smooth muscle-like cells (LAM cells) resulting in progressive cystic destruction of the lungs (1). Characteristic high-resolution computed tomography (HRCT) features are defined by multiple, thin-walled, well-defined lung cysts. Typically, there is no other significant pulmonary involvement, particularly no interstitial lung disease with the exception of possible features of multifocal micronodular pneumocyte hyperplasia in patients with tuberous sclerosis complex (TSC) (1). The diagnostic criteria for LAM are: 1) characteristic or compatible lung HRCT and lung biopsy fitting the pathological criteria for LAM; or 2) characteristic lung HRCT and any of the following: angiomyolipoma (kidney), thoracic or abdominal chylous effusion, lymphangioleiomyoma or lymph node involved by LAM, and definite or probable TSC (1).

In this case, we describe an unusual radiological presentation of LAM in a patient with a long follow-up of 33 years.

## Case report

In 1980, a 28-year-old woman presented with bilateral spontaneous pneumothorax. Ten days earlier she had a spontaneous abortion, but she was otherwise healthy and had no personal or family history of TSC. The patient was treated with bilateral chest tubes and left-sided surgical pleurectomy. Lung biopsy was performed, showing emphysematic lung tissue with tiny infiltrates of immature smooth muscle cells in the alveoli (i.e. LAM cells). Immunohistochemical receptor analysis was positive for estrogen and progesterone. A number of thin-walled dilated vessels were seen, filled up with proteinous fluid. The picture was compatible with sporadic LAM in an early stage. Oral progesterone treatment was started. During the following year, the patient suffered from dyspnea, chest pain, and frequent spontaneous pneumothoraces accompanied by small pleural effusions. Another lung biopsy was obtained showing similar pathological findings confirming the diagnosis of LAM (2). Chest x-rays showed varying degrees of diffuse reticulomicronodular infiltration and profuse hyperdiaphanous areas, indicating the presence of cystic lesions, thus supporting the diagnosis. The radiographs and the tissue samples unfortunately no longer exist.

In 1981 hysterosalpingo-oophorectomy was performed, resulting in improvement of the symptoms. Histological examination showed a 2-cm tumor in the omentum consisting of multiple small infiltrates of immature smooth muscle cells, interdigitating with fatty tissue. The histological pattern was very similar to the one found in the lung. In the uterus were found several typical fibroleiomyomas. Ovaries and tubes were normal. In 1982 a brief trial of tamoxifen momentarily worsened both the symptoms and chest x-ray. Progesterone treatment was intensified to intramuscular injections weekly, resulting in clinical improvement, ending of frequent pneumothoraces, and regression of the reticulomicronodular changes on chest x-ray. Clinical and radiological status was unchanged for many years at follow-up.

**Fig. 1:**
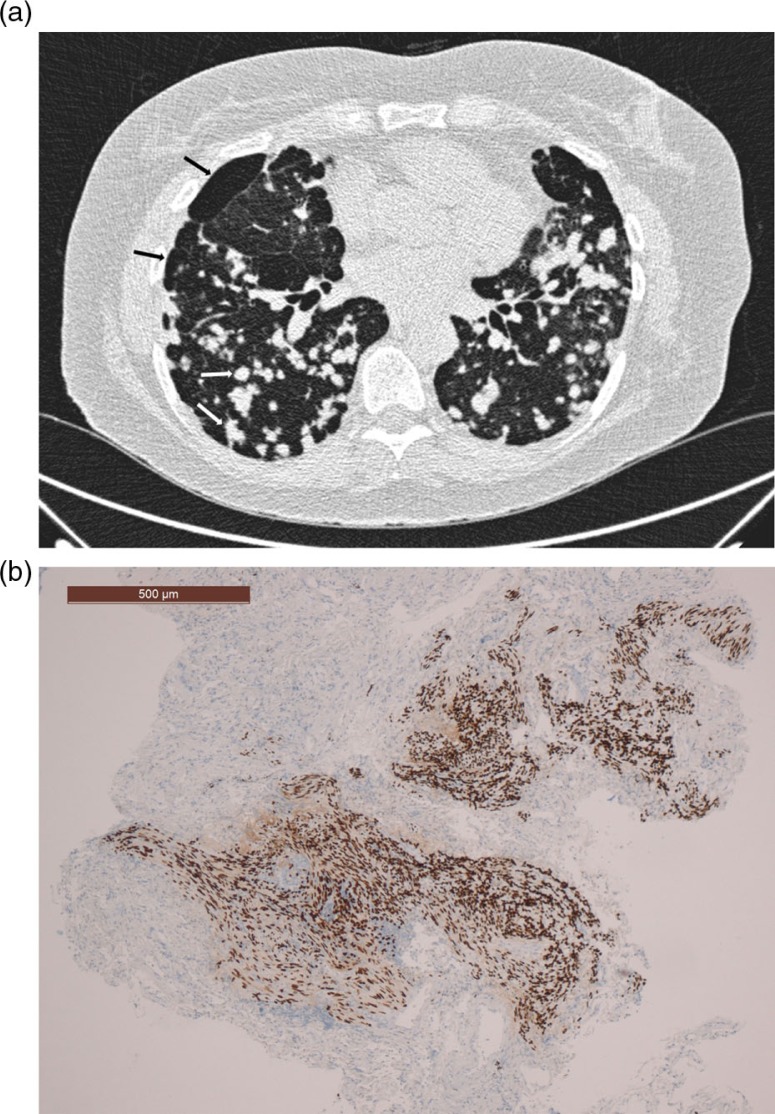
(a) HRCT showing nodules (white arrows) and cysts (black arrows). (b) Transbronchial biopsy of the lung (estrogen receptor immunohistochemical stain).

In 2010 HRCT was obtained for the first time, showing a few large thin-walled cysts and multiple coarse nodular changes distributed evenly in both lungs. The nodules varied in size from 3 to 2 cm. In 2012 a new HRCT showed slight increase in the size and number of nodules (Fig. 1a). Transbronchial biopsies showed a nodule with abnormal proliferation of spindle-shaped cells in fascicular streaks (Fig. 1b). These cells had elongated, hyperchromatic uniform nuclei. This area extended into the adjacent alveolar tissue without a distinct boundary. In the center of this area, there was a small cystic lesion. There was no evidence of interstitial inflammation, fibrosis, vasculitis, or granuloma formation. The alveolar septae were thickened in some areas. Immunohistochemical studies showed positive reaction of the spindle-shaped cells to estrogen, progesterone, desmin, vimentin, and alpha-smooth muscle actin. Human Melanoma Black 45 (HMB45), S-100, and MelanA were negative. It was concluded that the lesions were characteristic of LAM, and the diagnosis was confirmed.

Spirometry was normal when first measured at the time of diagnosis. During follow-up, the lung function has deteriorated to a mixed restrictive and obstructive pattern (forced expiratory volume in 1 sec (FEV1), 59% of predicted value; total lung capacity (TLC), 74%) with decreased diffusion capacity of the lung for carbon monoxide (DLco) of 53%. In 2012 echocardiography was normal.

Physical examination of the patient did not show signs of TSC (i.e. changes of the skin, eyes, or teeth). Furthermore, she had normal intellectual capacity and has never had seizures. The kidneys were not examined and genetic testing for TSC was not performed, thus TSC cannot be refuted.

## Discussion

Radiologically, cysts in the lungs and nodules may coexist and they could be a feature of LAM. Scattered nodules are more prevalent in TSC-associated LAM but were also described in sporadic LAM with associated multifocal micronodular pneumocyte hyperplasia.

In 2004, the World Health Organization classified LAM as a mesenchymal neoplasm (2). The pathologic appearance and the proliferative capacity are consistent with the hypothesis that LAM is a low-grade, destructive, metastasizing neoplasm (3). In our case, several features support this hypothesis. First is local proliferation of LAM cells in the lungs, leading to obliteration of the cysts and formation of multiple nodules. These nodules increased in size and numbers over time and radiologically resembled lung metastases. Second is invasion of the adjacent tissues, which is evident by the extension of the LAM nodules into the adjacent alveolar structure without a distinct boundary. Third, the histological findings of omental tumor and lung lesions were similar, compatible with abdominal localization of the same disease. A disease expressing the features of unlimited growth, local invasion, and distant metastasis is a malignancy, albeit in the case of LAM a low-grade malignancy that evolves over a period of decades in contrast to the majority of malignant diseases that have a shorter natural history.

Routine use of progesterone treatment is not recommended (1); however, in patients with rapid progression of symptoms or decline in lung function, intramuscular progesterone may be tried. Our patient received progesterone injections since 1982. It is unknown whether long-term treatment prompted excessive cell proliferation and the onset of nodules, but the observation that the cells were positive for hormone receptors might support this possibility. Recently, a case of sporadic LAM has been described, showing diffuse miliary micronodules and lung cysts evenly distributed in the lungs in a young woman (4). The follow-up of this patient should be interesting regarding whether the micronodules change into cystic lesions as in typical LAM, or if they evolve into macronodules as in our case.

In conclusion, we present an unusual case of LAM with profuse coarse lung nodules. We believe that this report might support the case for considering LAM a low-malignant neoplasm, with the possibility of an atypical and evolving radiological pattern.
